# A Delayed Presentation of a Sinus Venosus Atrial Septal Defect

**DOI:** 10.7759/cureus.91420

**Published:** 2025-09-01

**Authors:** Ashwath Elangovan, Keshav R Patel, Sina Salehi Omran

**Affiliations:** 1 Internal Medicine, University of Rochester School of Medicine and Dentistry, Rochester, USA; 2 Cardiology, University of Rochester Medical Center, Rochester, USA; 3 Interventional Cardiology, University of Rochester Medical Center, Rochester, USA

**Keywords:** adult congenital heart disease, advanced cardiac imaging, cox-maze, left atrial appendage clipping, partial anomalous pulmonary venous return (papvr), sinus venosus atrial septal defect, surgical asd repair

## Abstract

Sinus venosus atrial septal defects (ASDs) are rare congenital anomalies that result from an abnormality of the junction between the right atrium, superior vena cava (SVC), and pulmonary veins. This defect causes right-to-left shunting, which can lead to progressive right heart enlargement. We present a case of a 59-year-old man with a history of hypertension and hyperlipidemia who presented with dyspnea and newly diagnosed atrial flutter. Transthoracic echocardiography, transesophageal echocardiography, and CT imaging revealed a sinus venosus ASD with multiple anomalous pulmonary venous returns. The patient underwent successful surgical repair of the defect, which included ASD closure, augmentation of the SVC, and redirection of the anomalous pulmonary veins into the left atrium. This case underscores the importance of maintaining a high index of suspicion for congenital defects such as sinus venosus ASDs, even in adults, and the role of advanced imaging and timely surgical repair in these patients.

## Introduction

Sinus venosus atrial septal defects (ASDs) are rare congenital heart defects characterized by an abnormal connection between the junction of the superior vena cava (SVC), right atrium (RA), and pulmonary veins. Embryologically, the sinus venosus is a venous structure that becomes incorporated into the posterior right atrial wall. Abnormal or incomplete incorporation can leave patent connections between pulmonary veins and systemic venous circulation [1]. These defects, which account for about 5%-10% of ASDs, most commonly occur near the SVC and are often associated with anomalous pulmonary venous return [2]. Unlike secundum ASDs, which have a female predominance, sinus venosus ASDs show an approximately equal gender distribution [2]. The resultant left-to-right shunt can lead to progressive right-sided pressure and volume overload, paving the way for complications from right atrial and ventricular dilation [3].

While these are congenital defects, the timing of clinical presentation varies greatly between patients. This is largely because the hemodynamic impact of these shunts is multifactorial, with defect size being a major determinant of clinical outcomes [4]. When symptoms do occur, they commonly include exercise intolerance in the context of dyspnea or fatigue. Earlier detection and intervention are associated with better outcomes and reduction of long-term complications [2,5].

Echocardiography is commonly used to diagnose ASDs. While transthoracic echocardiography (TTE) is the first imaging modality used to evaluate most cardiac conditions, detecting sinus venosus defects on TTE can be challenging due to their posterior location [6]. In contrast, TEE is better suited for diagnosing these defects, as its positioning relative to the transducer allows for clearer visualization of posterior abnormalities [7]. Cardiac MRI and CT can further delineate the anatomy for surgical repair and detect anomalous pulmonary venous connections. 

We present a rare case of a 59-year-old man with sudden-onset dyspnea and fatigue, found to have a new-onset atrial flutter. A comprehensive cardiac evaluation revealed a sinus venosus defect with anomalous pulmonary venous return.

## Case presentation

**Figure 1:**
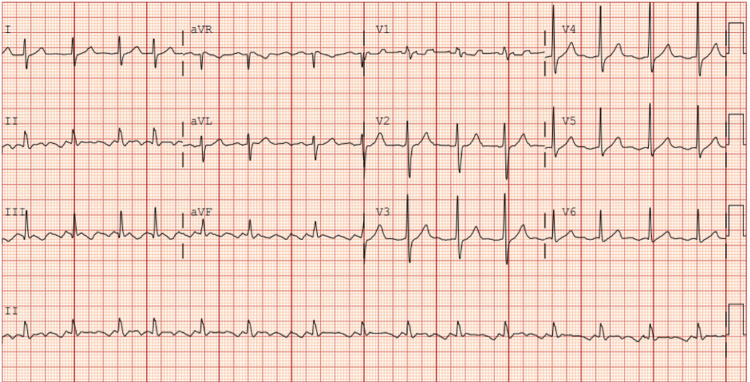
EKG EKG revealed typical atrial flutter with variable conduction.

A 59-year-old man with hypertension, hyperlipidemia, and moderate alcohol use presented to the clinic with a two-day history of dyspnea on exertion and fatigue. He was found to have new-onset atrial flutter (Figure 1). Notably, our patient underwent a stress TTE at the age of 55 years, which showed normal left and right ventricular size and function, normal estimated pulmonary artery pressures, and no obvious septal defects. However, the study was technically limited with suboptimal imaging windows, which likely contributed to the defect being missed. No follow-up TEE or cross-sectional imaging was pursued at that time. 

The patient was euvolemic on physical examination. Laboratory studies (Table 1) showed a hemoglobin level of 13.7 g/dL, a white blood cell count of 6,500 cells/uL, and a platelet count of 265,000 /uL. Renal function was normal with a creatinine of 0.99 mg/dL. Additional testing demonstrated a thyroid-stimulating hormone level of 0.94 mIU/L, ruling out hyperthyroidism.

**Table 1:** Laboratory values

	Value	Standard reference range
Hemoglobin	13.7 g/dL	13.5-17.5 g/dL
White blood cell	6,500 cells/uL	4,500-11,000 cells/uL
Creatinine	0.99 mg/dL	0.7-1.2 mg/dL
Thyroid-stimulating hormone	0.94 mIU/L	0.45-4.12 mIU/L

**Figure 2:**
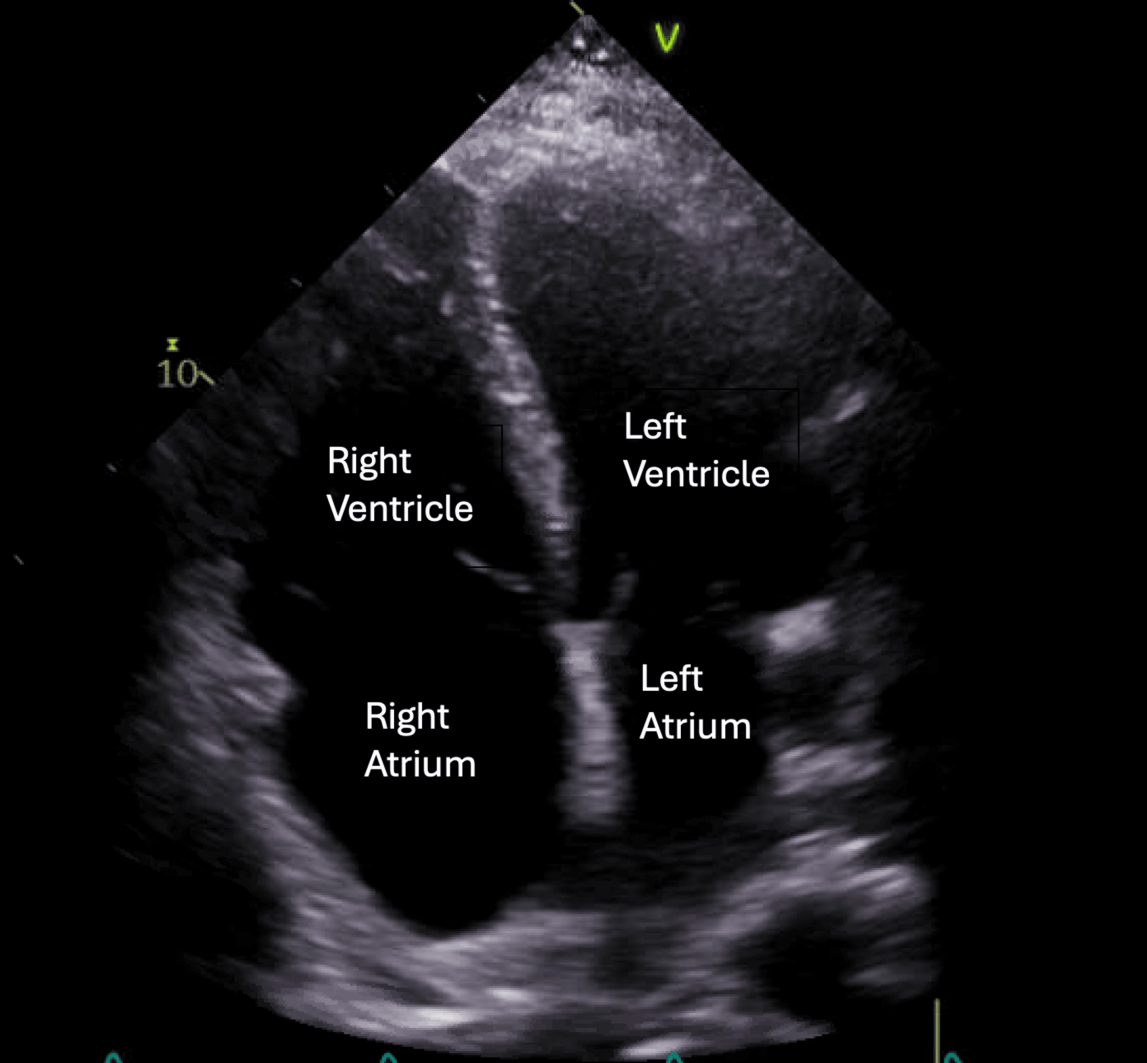
Transthoracic echocardiogram Apical four-chamber view revealed dilation of the right ventricle and right atrium, consistent with right-sided heart dysfunction. The septal motion abnormality observed is indicative of right ventricular pressure overload.

**Figure 3:**
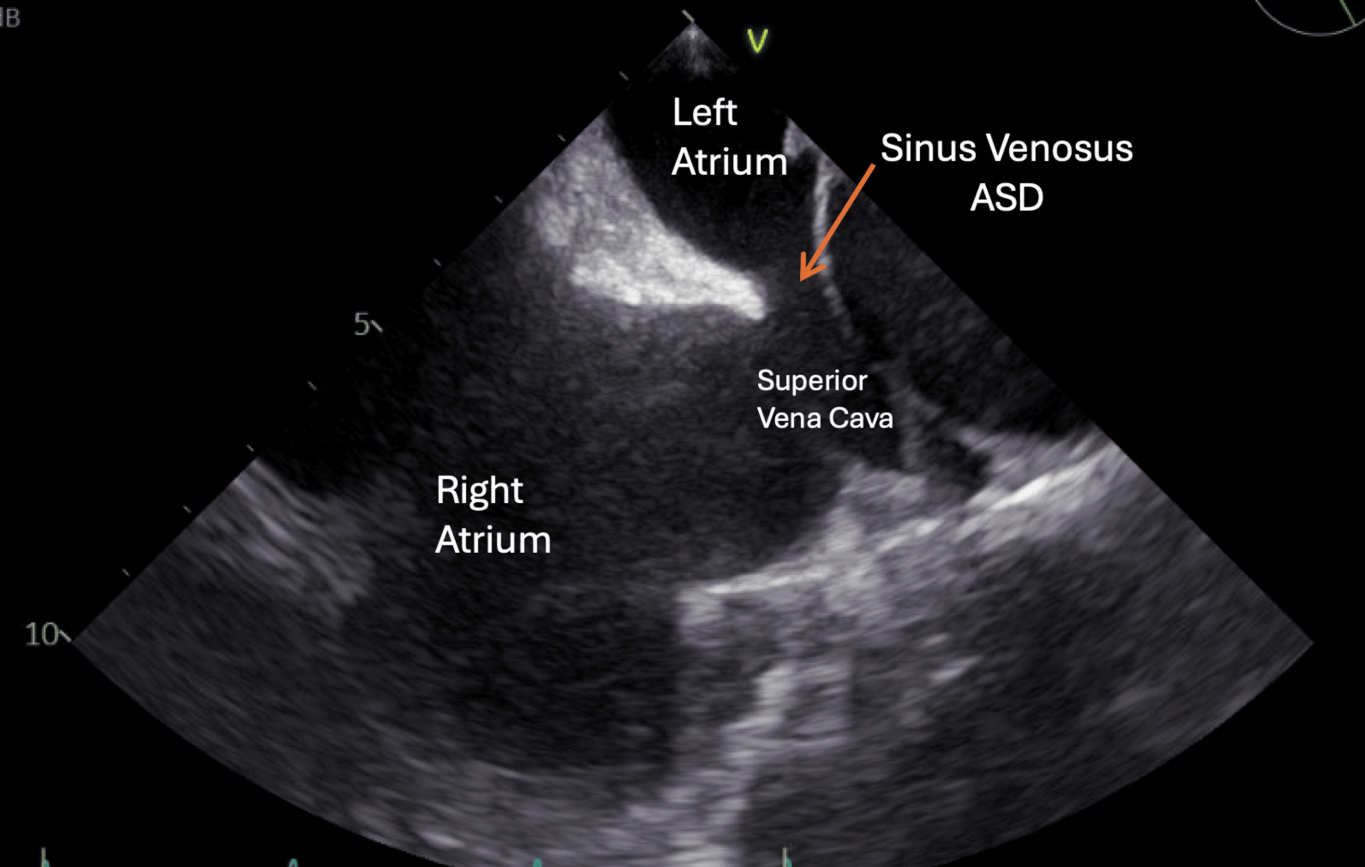
Transesophageal echocardiogram The bicaval view showed a sinus venosus ASD with associated PAPVR of right pulmonary veins to the SVC. ASD: Atrial septal defect; PAPVR: Partial anomalous pulmonary venous return; SVC: Superior vena cava

**Figure 4:**
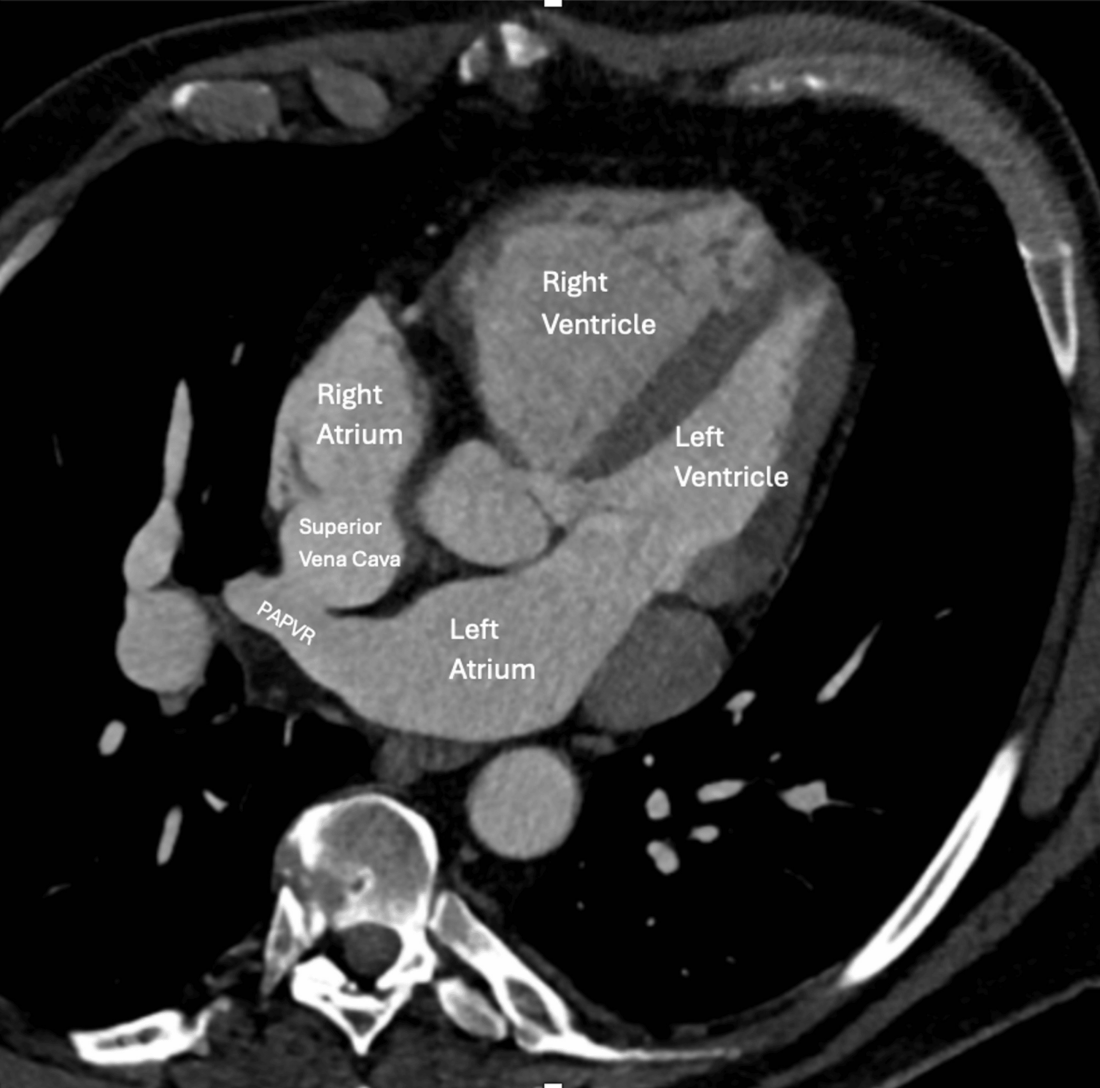
CT heart A contrast-enhanced CT of the heart showed four anomalous right pulmonary veins draining into the SVC, which was consistent with PAPVR. Additionally, a persistent left-sided SVC was noted, contributing to coronary sinus dilation. PAPVR: Partial anomalous pulmonary venous return; SVC: Superior vena cava

A TTE (Figure 2) revealed biatrial enlargement with septal motion abnormality due to the right ventricular pressure overload, consistent with severe right-sided heart enlargement with moderate pulmonary hypertension and coronary sinus dilation. A TEE (Figure 3) revealed a sinus venosus defect with multiple partial anomalous pulmonary venous returns (PAPVRs) of the right pulmonary vein to the SVC/sinus venosus defect. A CT scan revealed four anomalous right pulmonary veins and a persistent left-sided SVC that contributed to coronary sinus dilation (Figure 4). His pulmonary arteries were also enlarged. 

He was successfully cardioverted from atrial flutter to normal sinus rhythm during the TEE. At subsequent clinic visits, he was noted to have paroxysmal atrial fibrillation. 

Cardiac catheterization was performed to assess the intracardiac pressures, which revealed grossly normal findings (Table 2).

**Table 2:** Cardiac catheterization values Catheterization yielded normal right atrial and ventricular pressures, along with normal pulmonary artery pressure and pulmonary vascular resistance. He also had angiographically normal coronary arteries.

Parameter	Value	Normal range
Right atrium pressure	5 mmHg	2-8 mmHg
Right ventricle pressure	30/5 mmHg	Systolic: 15-30 mmHg, diastolic: 2-8 mmHg
Pulmonary artery pressure	29/12 mmHg	Systolic: 15-30 mmHg, diastolic: 8-15 mmHg
Pulmonary capillary wedge pressure	11 mmHg	6-12 mmHg
Aortic pressure	134/82 mmHg	Systolic: 90-140 mmHg, diastolic: 60-90 mmHg
Pulmonary vascular resistance	0.4 Wood units	0.25-1.5 Wood units
Systemic vascular resistance	19.6 Wood units	11-15 Woods Units
Cardiac output	5.1 L/min	
Cardiac index	2 L/min	
Pulmonary artery pulsatility index	3.4 mmHg/mmHg	2-5 mmHg/mmHg

In addition to the pressures, oxygen saturation data were also collected (Table 3). 

**Table 3:** Oxygen saturation values Oxygen saturations were measured from the SVC, IVC, RA, RV, and systemic circulation. Elevated SVC and RA saturations reflect anomalous pulmonary venous return and lead to overestimation of mixed venous O₂ content. For shunt calculation, the IVC saturation was used, yielding a corrected Qp/Qs of 2.9. IVC: Inferior vena cava; O₂: Oxygen; Qp/Qs: Pulmonary-to-systemic blood flow ratio; RA: Right atrium; RV: Right ventricle; SVC: Superior vena cava

	Saturation
Superior vena cava	93%
Inferior vena cava	64%
Right atrium	93%
Right ventricle	84%
Arterial	93%

The patient underwent surgical repair of the PAPVR defect and sinus venosus ASD defect. The autologous pericardium was used to close the ASD and augment the SVC, and the anomalous pulmonary veins were baffled into the left atrium. Additionally, during the surgery, he underwent clipping of the left atrial appendage and a Cox-Maze procedure to treat the atrial fibrillation/flutter and reduce the risk of recurrence and complications.

## Discussion

This case highlights the diagnostic challenges associated with congenital heart defects. This patient, at age 55 years, had a normal stress echocardiogram, but years later, he presented with severe right heart enlargement and moderate pulmonary hypertension. This progression captures how congenital heart defects, such as sinus venosus ASD with PAPVR, can remain asymptomatic for decades and then have a sudden manifestation with clinically significant consequences. While an adult diagnosis of congenital ASDs has been reported, a progression this rapid following a previously normal echocardiogram is rare and not commonly described in literature [8].

As observed in this case, the effects of the sinus venosus ASD and PAPVR have a variety of clinical consequences, ranging from severe right-sided heart failure to isolated arrhythmias. Hemodynamically, the left-to-right shunt leads to progressive right-sided volume and pressure overload, which in this case culminated in right-heart enlargement and dysfunction. 

This case highlights the importance of understanding shunting physiology. Using the values from Table 3, the typical mixed venous saturation equation ${Mixed\ venous}_{sat}=\ \frac{\ (3\times({\rm SVC}_{sat})\ +\ {\rm IVC}_{sat})}{4}$ would result in an erroneous pulmonary-to-systemic blood flow ratio (Qp/Qs) of 0.7, indicating a right-to-left shunt and potentially precluding this patient from an ASD closure. However, it is crucial to consider that SVC saturation is falsely elevated due to anomalous pulmonary vein return. The RA, due to its high sampling location, is falsely elevated for similar reasons; therefore, we ultimately decided to use the inferior vena cava saturation as the mixed venous saturation. This process revealed a true Qp/Qs of 2.9, indicative of a left-to-right shunt. This diagnostic pitfall, an overestimated SVC oxygen saturation due to PAPVR, is not commonly highlighted in existing literature and could lead to inappropriate exclusion from surgical repair. While invasive hemodynamic data were used in this case for surgical planning purposes, non-invasive techniques such as Doppler echocardiography or cardiac MRI can also be used to quantify shunts. Future cases may benefit from a comparison of calculations between the different modalities. 

This patient presented with atrial flutter, which is associated with atrial enlargement affecting the conduction system. Atrial arrhythmias (AAs) have an increased incidence in patients with ASDs. In patients who have not undergone surgical repair, the incidence of AAs is around 10%-20% in patients under 40 years of age, but with increasing age, the incidence of AAs rises [9]. He was cardioverted during the TEE, but he remained intermittently in atrial fibrillation afterward. The decision for the left atrial appendage clip and Cox-Maze procedure was guided by a 2a recommendation in performing concurrent ablation in patients undergoing other planned cardiac surgery [10]. For our patient, we elected to continue amiodarone to maintain sinus rhythm. We additionally chose to continue apixaban and will consider discontinuation once ambulatory heart rate monitoring has been completed. 

Advanced imaging played a crucial role in this patient’s diagnosis. TTE was able to identify abnormalities consistent with right heart enlargement. However, sinus venosus ASDs are technically difficult to detect on TTE due to their posterior location near the SVC/RA junction, which is often outside the optimal imaging plane. This is likely why the original transthoracic echocardiogram was interpreted as normal, given that it was a limited study with suboptimal acoustic windows. In contrast, TEE places the transducer in closer proximity to the posterior heart and was required to establish the presence of a sinus venosus ASD, PAPVR, and a dilated coronary sinus in this case. CT imaging further revealed four anomalous pulmonary veins and a persistent left-sided SVC, which led to coronary sinus dilation. This illustrates the role of advanced imaging modalities such as TEE and CT in treatment planning when initial echocardiograms are inconclusive [8]. In addition to defining the cardiac anatomy, preoperative coronary artery evaluation also allows the surgical team to proceed with isolated ASD and PAPVR repair without the need for concomitant coronary revascularization. He eventually underwent a successful surgical intervention, which included closure of the ASD, redirecting the anomalous pulmonary veins into the left atrium, and using pericardium to augment the SVC. The surgical approach was chosen in this case, as our patient was deemed a viable surgical candidate. Surgery is preferred in repairing ASDs, but for patients who are not favorable surgical candidates, percutaneous repair is an option [4,11,12]. The overall prognosis for patients who undergo surgical management of sinus venosus ASDs is excellent, with both low operative mortality and encouraging long-term results [13].

Our report is limited by the absence of a long-term follow-up for this patient, which would have allowed for tracking of post-surgical right ventricular remodeling and arrhythmia recurrence. Additionally, the initial echocardiogram four years before was a limited TTE with suboptimal acoustic windows, so the timing and progression of the defect remain uncertain. 

## Conclusions

Congenital heart defects may not be diagnosed until late adulthood due to the absence of clinically significant signs and symptoms. This case emphasizes the importance of a high index of suspicion for shunting in patients with right-sided heart enlargement. In this patient, PAPVR led to falsely elevated oxygen saturations in the SVC. This is important to consider in the context of shunt calculations, as they can affect candidacy for ASD closure. This case also demonstrates the utility of advanced imaging modalities that are helpful in further delineating complex cardiac anatomy.

## References

[1] Anderson RH, Brown NA, Webb S (2002). Development and structure of the atrial septum. Heart.

[2] Webb G, Gatzoulis MA (2006). Atrial septal defects in the adult: recent progress and overview. Circulation.

[3] Sommer RJ, Hijazi ZM, Rhodes JF (2008). Pathophysiology of congenital heart disease in the adult: part III: complex congenital heart disease. Circulation.

[4] Celermajer DS (2018). Atrial septal defects: even simple congenital heart diseases can be complicated. Eur Heart J.

[5] Attenhofer Jost CH, Connolly HM, Danielson GK (2005). Sinus venosus atrial septal defect: long-term postoperative outcome for 115 patients. Circulation.

[6] Crystal MA, Al Najashi K, Williams WG, Redington AN, Anderson RH (2009). Inferior sinus venosus defect: echocardiographic diagnosis and surgical approach. J Thorac Cardiovasc Surg.

[7] Pascoe RD, Oh JK, Warnes CA, Danielson GK, Tajik AJ, Seward JB (1996). Diagnosis of sinus venosus atrial septal defect with transesophageal echocardiography. Circulation.

[8] Qiu JK, Bamira D, Vainrib AF, Latson LA, Halpern DG, Chun A, Saric M (2022). Multimodality imaging of sinus venosus atrial septal defect: a challenging diagnosis in adults. CASE (Phila).

[9] Chubb H, Whitaker J, Williams SE, Head CE, Chung NA, Wright MJ, O'Neill M (2014). Pathophysiology and management of arrhythmias associated with atrial septal defect and patent foramen ovale. Arrhythm Electrophysiol Rev.

[10] Joglar JA, Chung MK, Armbruster AL (2024). 2023 ACC/AHA/ACCP/HRS guideline for the diagnosis and management of atrial fibrillation: a report of the American College of Cardiology/American Heart Association Joint Committee on Clinical Practice Guidelines. Circulation.

[11] Baruteau AE, Hascoet S, Malekzadeh-Milani S (2023). Transcatheter closure of superior sinus venosus defects. JACC Cardiovasc Interv.

[12] Stout KK, Daniels CJ, Aboulhosn JA (2019). 2018 AHA/ACC guideline for the management of adults with congenital heart disease: executive summary: a report of the American College of Cardiology/American Heart Association Task Force on Clinical Practice Guidelines. Circulation.

[13] El-Andari R, Moolla M, John K (2024). Outcomes following surgical repair of sinus venosus atrial septal defects: a systematic review and meta-analysis. J Am Heart Assoc.

